# Early diagnosis of hollow viscus injury using intestinal fatty acid–binding protein in blunt trauma patients

**DOI:** 10.1097/MD.0000000000006187

**Published:** 2017-03-10

**Authors:** Shokei Matsumoto, Kazuhiko Sekine, Hiroyuki Funaoka, Tomohiro Funabiki, Masayuki Shimizu, Kei Hayashida, Mitsuhide Kitano

**Affiliations:** a Department of Trauma and Emergency Surgery, Saiseikai Yokohamashi Tobu Hospital, Yokohama, Japan; b Department of Emergency Medicine, Saiseikai Central Hospital, Minato-ku, Tokyo, Japan; c DS Pharma Biomedical Co., Ltd., Osaka, Japan.

**Keywords:** blunt abdominal trauma, diagnosis, hollow viscus injury, intestinal fatty acid binding protein

## Abstract

A delay in diagnosing hollow viscus injury (HVI) causes an increase in mortality and morbidity. HVI remains a challenge to diagnose, and there is no specific diagnostic biomarker for HVI. We evaluated the utility of intestinal fatty acid–binding protein (I-FABP) in diagnosing HVI in blunt trauma patients. Within a 5-year period, 93 consecutive patients with clinically suspected HVI at our trauma center were prospectively enrolled. The diagnostic performance of I-FABP for HVI was compared with that of other various parameters (physical, laboratory, and radiographic findings). HVI was diagnosed in 13 patients (14%), and non-HVI was diagnosed in 80 patients (86%). The level of I-FABP was significantly higher in patients with HVI than in those with non-HVI (*P* = 0.014; area under the curve, 0.71). The sensitivity, specificity, positive predictive value, and negative predictive value were 76.9%, 70.0%, 29.4%, and 94.9%, respectively (*P* = 0.003). However, all other biomarkers were not significantly different between the groups. Presence of extraluminal air, bowel wall thickening on computed tomography (CT), and peritonitis signs were significantly higher in patients with HVI (*P* < 0.05). Of 49 patients (52.7%) who had a negative I-FABP and negative peritonitis signs, none developed HVI (sensitivity, 100%; negative predictive value, 100%). This is the first study that demonstrated the diagnostic value of a biomarker for HVI. I-FABP has a higher negative predictive value compared to traditional diagnostic tests. Although the accuracy of I-FABP alone was insufficient, the combination of I-FABP and other findings can enhance diagnostic ability.

## 1 Introduction

Delayed diagnosis of hollow viscus injury (HVI) significantly increases mortality and morbidity in blunt trauma patients.^[1–3]^ Although medical technology has improved in recent years, diagnosis management in patients with a suspected HVI has not progressed, and exploratory laparotomy is still the most reliable method for diagnosis. However, a negative or nontherapeutic laparotomy has a high risk of complications.^[4,5]^ HVIs are frequently found in patients who undergo laparotomy for hemostasis of other solid organ injuries, but the chances to directly diagnose HVI via laparotomy are decreasing as nonoperative management is becoming more popular. Radiological computed tomography (CT) and diagnostic peritoneal lavage may be considered useful, but they are costly, invasive, and require significant expertise. These methods all have associated problems, and a simple cost-effective and noninvasive diagnostic tool is needed to ensure prompt diagnosis.

At present, there are no specific biomarkers for diagnosing HVI. Although white blood cell count (WBC) is commonly elevated by physical stress in trauma patients, a persistently elevated WBC may suggest a potential HVI based on observation in the first 24 hours.^[6]^ However, even a diagnostic delay of <8 hours results in a worse prognosis.^[1]^ Many studies have reported the diagnostic value of CT for HVI, but there are few studies on the diagnostic value of biomarkers for HVI. Recently, intestinal fatty acid–binding protein (I-FABP) has been suggested as a new biomarker to diagnose intestinal disease.^[7–12]^ It was also reported to be associated with severe abdominal injuries.^[13–16]^ I-FABP is a small (14–15 kDa), cytosolic, water-soluble protein that comprises up to 2% of the cytoplasmic protein content of the mature enterocyte, and is abundant in bowel mucosa. If the intestinal mucosal tissue is injured, I-FABP is rapidly released into the bloodstream. However, no study has examined the diagnostic value of I-FABP for HVI. We hypothesized that I-FABP adds to the diagnostic arsenal in the setting of blunt abdominal trauma. This study was undertaken to evaluate the utility of I-FABP, sampled at the time of admission, in the early diagnosis of HVI.

## 2 Methods

This study was approved by the local ethics committee of Saiseikai Yokohamashi Tobu Hospital. The study was conducted at the Emergency and Trauma Center of Saiseikai Yokohamashi Tobu Hospital, a tertiary-care hospital in Yokohama, Japan, from January 2010 to December 2014. All consecutive blunt trauma patients with suspected clinical HVI who underwent abdominal and pelvic CT at our trauma department were prospectively enrolled. Clinical findings suggestive of HVI were at least one of the following: abdominal pain, abdominal distention, a mechanism associated with the abdominal region, and abnormal skin on the abdomen. These conditions were identified by an attending acute care surgeon at an initial evaluation. We excluded patients younger than 18 years, and patients with refractory shock who could not undergo a CT.

### 2.1 Study design and sample collection

After enrollment, the following items were recorded for each patient: age, sex, vital signs, physical abdominal examinations (tenderness and peritoneal signs), radiologic findings, and routine laboratory test results. Peritoneal signs were defined as rebound tenderness and guarding. After the initial assessment, blood samples were taken to investigate I-FABP and other biomarkers. The diagnostic performance of I-FABP for HVI was compared with that of other parameters, including physical, laboratory, and radiographic findings.

All patients were treated according to the Advanced Trauma Life Support (ATLS) Course guidelines,^[17]^ without interference by the research team. The attending Japanese board-certified surgeon determined the operative indication in a comprehensive manner, taking the CT findings, physical examination results, and laboratory results into consideration. According to the clinical and surgical results, the diagnosis of each patient was retrospectively classified as either HVI or non-HVI.

### 2.2 Radiographic imaging and evaluation

All ultrasonography (US) examinations were performed using a focused assessment with sonography for trauma (FAST) by Japanese board-certified attending emergency physicians. A US imaging unit (Viamo, Toshiba, Tokyo, Japan) with a 5.0-MHz convex probe was used. All CT scans were performed using 64 multidetector CT scanners (Aquilion CT scanner; Toshiba, Tokyo, Japan) at the initial management. Intravenous contrast medium (iohexol, Omnipaque 300; Daiichi Sankyo, Tokyo, Japan) was used in all patients unless contraindicated. CT images were reviewed retrospectively, with agreement between an attending radiologist and an experienced faculty emergency radiologist. The following CT features were assessed: extraluminal air, free fluid, bowel wall thickening, contrast extravasation, and the presence of solid organ injury. These findings were based on a study by Fakhry et al.^[2]^

### 2.3 Laboratory analysis

The I-FABP assay is currently in clinical development and has not yet been used in a clinical setting. For the I-FABP assay, samples were stored at −20°C until analysis. I-FABP was measured at the DS Pharma Biomedical Center in Osaka, Japan, at a later date. Thus, I-FABP did not impact the decision-making in this study. I-FABP serum levels were quantified using synthetic regional peptides and a recombinant I-FABP assay (Dainippon Sumitomo Pharma; Osaka, Japan). The serum I-FABP level in the healthy volunteers was 1.1 ± 0.9 ng/mL, ranging from 0.1 to 2.0 ng/mL.^[18]^ Other biomarkers were as follows: WBC, hemoglobin (HGB), lactate, and amylase (AMY). All other biomarkers were measured immediately in serum using commercially available assays at Saiseikai Yokohamashi Tobu Hospital. The normal ranges of the markers used in this study were as follows: WBC, 3.50 to 8.50 cells/mm^3^ × 1000; HGB (males), 13.8 to 17.2 g/dL; HGB (females), 12.1 to 15.1 g/dL; lactate, 4 to 16 mg/dL, and AMY, 40 to 126 IU/L.

### 2.4 Statistical analyses

Analysis was performed using the *χ*^2^ test and the Mann–Whitney *U* test, as appropriate. To identify the diagnostic value of these biomarkers for HVI, receiver operator characteristic (ROC) curves were constructed, and the area under the curve (AUC) was calculated. The best cutoff points were defined as the maximum sum of sensitivity and specificity. A value of *P* < 0.05 was considered statistically significant.

## 3 Results

### 3.1 Patient demographics, characteristics, and outcomes

Ninety-three patients were enrolled during the study period. During this study, a total of 3460 blunt trauma patients attended the emergency department, and 2024 patients underwent abdominal CT (including 1912 patients who underwent whole-body CT). Patient characteristics and outcomes are shown in Table 1. Patients in the sample were a mean of 45 years, and the majority were male (72.0%). The median Injury Severity Score (ISS) and the rate of the abdominal Abbreviated Injury Score (AIS) ≥3 were 17 (interquartile range: 9.0–27.0) and 31%, respectively. These patients were involved in motor vehicle accidents (79.6%), had fallen (16.1%), or had other causes of injury (4.3%). Overall, the rate of exploratory surgery was 38.7% (n = 36), and the mortality within 28 days was 6.5% (all deaths not related to HVI). None of the patients had undergone diagnostic peritoneal lavage. Of the patients who underwent exploratory surgery, 25% (n = 9) received diagnostic laparoscopy. Among them, 4 patients (44%) converted to laparotomy to repair the HVI. Besides diagnostic laparoscopy, there were 4 (4.3%) unnecessary laparotomies and no delayed laparotomy in this study. The other 27 patients (29.0%) underwent angiographic embolization. Among these patients, 14 patients (51.9%) received angiographic embolization for hemostasis of pelvic fractures. Concomitant injuries included liver injury and pelvic fracture in approximately 20% of patients.

**Table 1:**
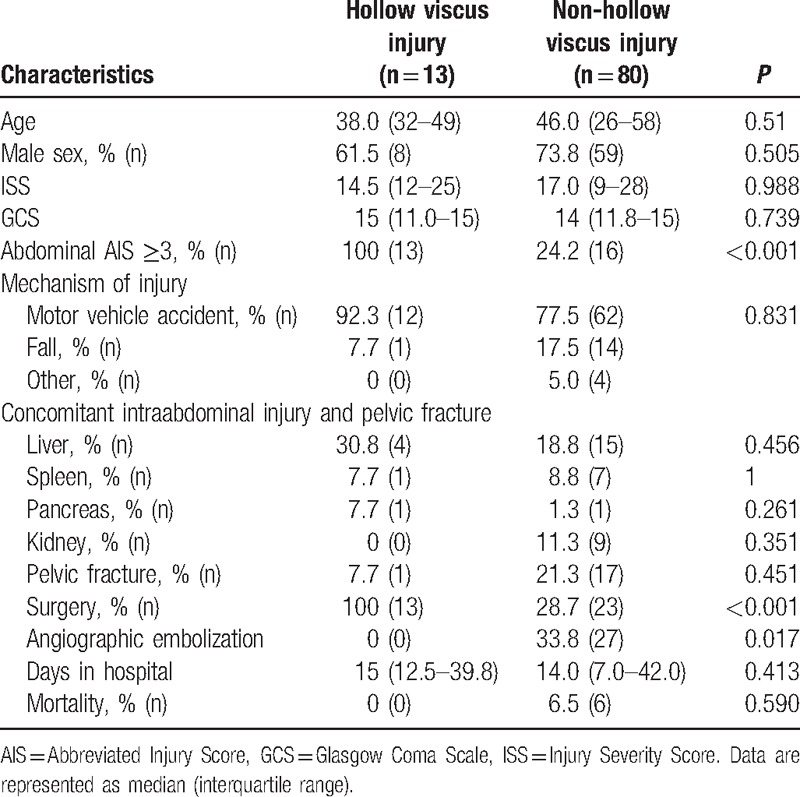
Patient characteristics and outcomes with hollow viscus injury and nonhollow viscus injury.

HVI was diagnosed in 13 patients (14%), and non-HVI was diagnosed in 80 patients (86.0%). In the HVI group, 7 patients (53.8%) had isolated HVI without concomitant intraabdominal injuries. The rate of operative exploration and AIS ≥3 was significantly higher in patients with HVI than that in patients with non-HVI (100% vs. 28.7%, *P* < 0.001; 100% vs. 24.2%, *P* < 0.001, respectively). The rate of angiographic embolization was significantly lower in patients with HVI than in patients with non-HVI (0% vs. 33.8%, *P* < 0.017). There were no significant differences between the other characteristics and in-hospital mortality between the 2 groups.

### 3.2 Vital signs, clinical presentations, and physical findings

The diagnostic findings are shown in Table 2. For vital signs, the respiratory rate was not used because it was difficult to measure and might be inaccurate. There was no significant difference in vital signs between the 2 groups. The most common presentation upon physical examination was tenderness (38.7%), followed by peritonitis sign (19.4%). The rate of both findings was significantly higher in patients with HVI than that in patients with non-HVI (*P* < 0.05). Peritonitis sign was strongly associated with HVI (odds ratio [OR] 17.75; 95% confidence interval [CI]: 4.53–69.63).

**Table 2:**
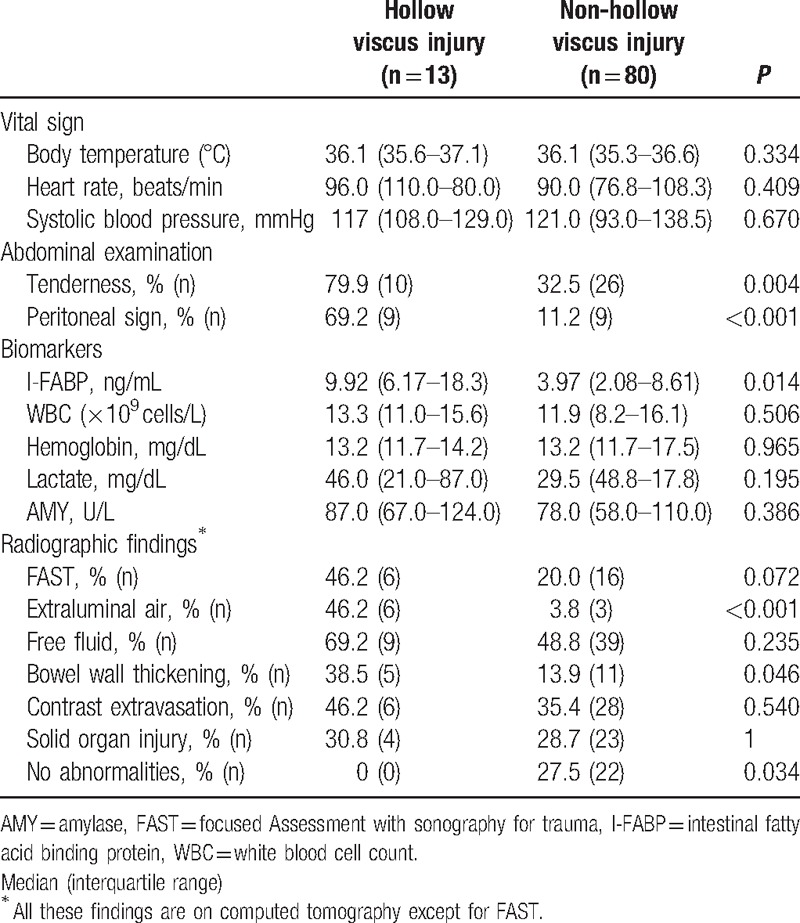
Diagnostic findings with hollow viscus injury and non-hollow viscus injury.

### 3.3 Laboratory findings

All biomarker levels except HGB were abnormal in both groups. Only the I-FABP level was significantly higher in patients with HVI than in those without HVI (9.92 [6.17–18.3] ng/mL vs. 3.97 [2.08–8.61] ng/mL, *P* = 0.014). However, all other biomarker levels did not significantly differ between groups. ROC analysis showed that the AUC was highest for I-FABP (AUC = 0.71) in diagnosing HVI. Using the I-FABP best cutoff value (6.2 ng/mL), the sensitivity, specificity, positive predictive value, and negative predictive value were 76.9%, 70.0%, 29.4%, and 94.9%, respectively (OR 7.78; 95% CI: 1.97–30.79).

### 3.4 Radiological findings

The rate of positive FAST did not differ significantly between the 2 groups. Intravenous contrast media were used for all patients. The most common finding on CT was free fluid (51.6%). Of those with free fluid, only 18.8% had HVI. The presence of extraluminal air and bowel wall thickening was significantly higher in patients with HVI than in those without (OR 22.00; 95% CI: 4.50–107.59; OR 3.86; 95% CI: 1.07–13.98, respectively). The specificities were high (96.3%, 86.1%, respectively), but the sensitivities were low (46.2%, 38.5%, respectively). All patients with HVI had some kind of abnormal findings on CT, and the rate of no abnormal findings on CT was significantly lower in patients with HVI (*P* = 0.034). The lack of abnormal findings on CT had 100% sensitivity and a negative predictive value, but specificity was 27.5%. There were no significant differences in the other radiographic findings (free fluid, contrast extravasation, and solid organ injury) between the 2 groups.

### 3.5 Combination of I-FABP and other findings

**Figure 1:**
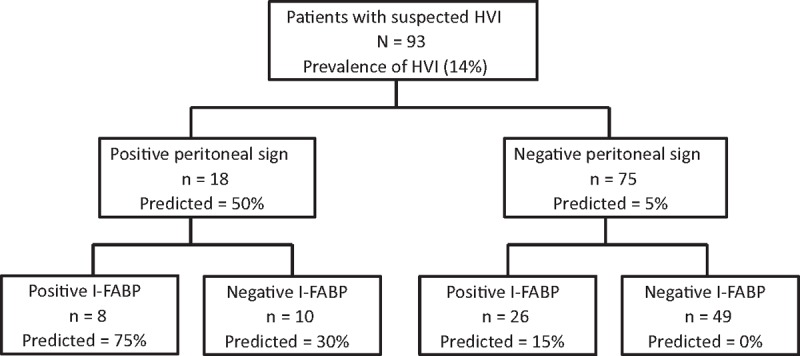
Decision tree using I-FABP and physical examination for the management of patients with suspected HVI. HVI = hollow viscus injury, I-FABP = intestinal fatty acid–binding protein.

**Figure 2:**
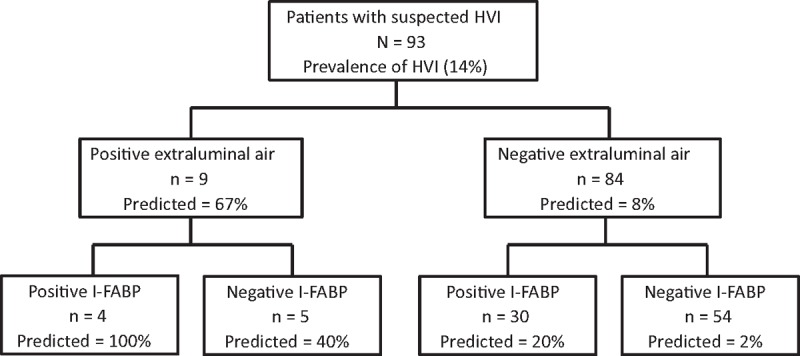
Decision tree using I-FABP and extraluminal air on computed tomography for the management of patients with suspected HVI. HVI = hollow viscus injury, I-FABP = intestinal fatty acid–binding protein.

The sensitivity, specificity, and positive and negative predictive value of I-FABP, peritonitis sign, and CT findings are shown in Table 3. Because the accuracy of I-FABP alone was insufficient, we hypothesized that the combination of I-FABP with other findings was better. To improve the sensitivity, I-FABP was combined with peritonitis sign. Figure 1 shows the relationship between I-FABP and peritonitis sign findings for predicting HVI. Among this study population, 52.7% of patients (n = 49) had a negative I-FABP (<6.2 mg/dL) and a negative peritonitis sign, and none of them developed HVI (sensitivity, 100% and negative predictive value, 100%; *P* < 0.001). Similarly, to improve specificity, I-FABP was combined with extraluminal air findings on CT. Figure 2 shows the relationship between I-FABP and extraluminal air findings on CT for predicting HVI. In this study population, 4.3% (n = 4) of patients had a positive I-FABP (>6.2 mg/dL) and extraluminal air on CT, and all of these patients developed HVI (specificity, 100% and positive predictive value, 100%; *P* < 0.001).

**Table 3:**
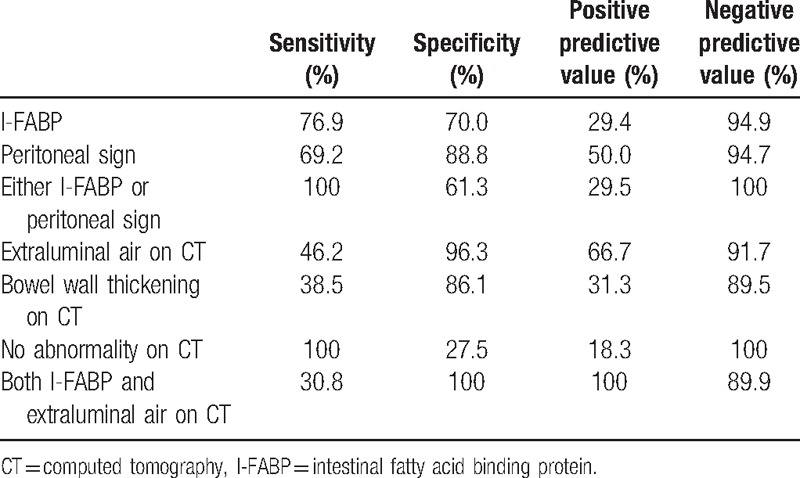
Sensitivity, specificity, and positive and negative predictive values of I-FABP, peritoneal sign, and CT findings in detecting hollow viscus injury.

## 4 Discussion

This is the first study that demonstrates the diagnostic value of I-FABP and its value as a biomarker for HVI. HVI in blunt trauma patients is challenging to diagnose. The incidence of morbidity and mortality increases with time to operative intervention.^[1]^ Currently, nonoperative management has become the treatment standard for mild solid organ injury that is hemodynamically stable, and it increases the risk of HVI, which always requires surgery. However, there is currently no reliable method to diagnose HVI in blunt trauma patients. A revised management strategy and new techniques are required to assist in diagnosing HVI. The largest study of 275,557 blunt trauma patients reported that current HVI diagnostic approaches lacked sensitivity.^[2]^ However, the present study demonstrated that a combination of peritonitis and I-FABP had 100% sensitivity for diagnosing HVI. This means that for patients with negative I-FABP and negative peritonitis signs, HVI can be ruled out at the time of admission. Patients without other injuries can be discharged immediately.

Although there are many studies on the diagnosis of HVI, most are retrospective studies, and there are few studies to date that combine CT findings, laboratory findings, and physical findings to diagnose HVI. It is difficult to study prospectively because blunt HVI is relatively rare and the incidence is <1%.^[19]^ In the present study, HVI occurred in only 0.6% of patients with abdominal CT. The low incidence and lack of experience make it difficult to diagnose HVI. Some studies reported that solid organ injuries, seat belt sign, and injury mechanisms related to the abdomen are associated with an increased risk of HVI.^[2,20,21]^ Thus, the probability of diagnosing HVI in this study increased by 14.0% in this patient population. Surgeons should perform a thorough examination of blunt trauma patients who have these findings that may suggest HVI.

For blunt trauma patients, the presence of plasma biomarkers has long served as a diagnostic tool for organ injuries, especially of the heart and liver.^[22,23]^ Alanine aminotransferase and troponin I are used extensively, since quickly measuring these biomarkers in the plasma of emergency department patients can prove extremely useful for detecting heart and liver injuries. Additionally, heart-type FABP is widely used to treat acute myocardial infarction. Using the same mechanism as heart and liver injury, I-FABP, which is found exclusively in the intestinal mucosa, could possibly be released into the bloodstream following tissue injury.^[24]^ Therefore, we aimed to improve the diagnosis of HVI using I-FABP. I-FABP is the only biomarker that is bowel membrane–specific, and it was also the top diagnostic performer in this study.

The diagnostic accuracy of I-FABP alone was insufficient for patients with HVI. I-FABP can be elevated in other types of bowel disease, such as small bowel obstruction, mesenteric ischemia, acute enterocolitis, Crohn disease, ulcerative colitis, and necrotizing enterocolitis.^[7,11,12,25–27]^ Besides these factors, I-FABP can increase the false-positive rate in trauma patients. First, hemorrhagic shock and systemic inflammatory response are known to cause intestinal mucosal damage.^[28]^ I-FABP levels increase in severe trauma patients without HVI.^[13–16]^ Haan et al reported that the level of I-FABP was significantly related to the interleukin 6 level and the presence of shock. Second, I-FABP can be elevated in severe abdominal trauma to the diaphragm, liver, and spleen, without HVI.^[13,15,16]^ I-FABP levels are significantly higher in patients with abdominal AIS ≥3 than in those with abdominal AIS <3. This might indicate that a direct blow to the abdomen affects I-FABP levels. I-FABP might also be released from a less severely injured bowel that does not require surgery, such as submucosal hematoma, bowel bruise, or a small serosal tear. In this present study, true bowel findings in patients with nonsurgical management were unavailable. These patients may have had a self-limited, unidentified bowel injury during their disease course. Therefore, surgeons should consider these false-positives when using I-FABP. We believe that I-FABP can play a more decisive role in hemodynamically stable blunt trauma patients.

Currently, CT is the most commonly used modality for diagnosing abdominal trauma, and it is regarded as highly accurate in identifying solid-organ injuries. However, the usefulness of CT for diagnosing HVI in blunt trauma patients remains controversial. With the development of the multidetector CT, HVI diagnosis may be more accurate. In a recent study that used multidetector CT for surgically important bowel and mesenteric injury after blunt trauma, sensitivities and specificities ranged from 87% to 95%, and 48% to 84%, respectively.^[29]^ In the Eastern Association for the Surgery of Trauma (EAST) study of 3258 trauma patients with CT, 13% of blunt trauma patients with perforated small bowel injury had no abdominal findings.^[2]^ In the present study, a high multidetector CT was used, and the accuracy was relatively high compared to previous studies. Extraluminal air on CT had a low sensitivity and a high specificity (46.2% and 96.3%, respectively). Although extraluminal air is classically a finding associated with HVI that requires surgery, recent studies reported that extraluminal air has a high false-positive rate (29%–61%) in blunt trauma patients.^[2,29–31]^ Marek et al^[30]^ demonstrated that free fluid, seat belt sign, or radiographic findings that suggest HVI in the presence of extraluminal air are highly predictive of HVI. The present study demonstrated that I-FABP has a high predictive value for diagnosing HVI in blunt trauma patients with extraluminal air on CT. Combined I-FABP and CT findings might be useful in diagnosing HVI.

CT is a useful modality for trauma management, but a plasma biomarker for trauma patients has various advantages compared to CT. First, CT is expensive and not uniformly available worldwide. Second, there are absolute or relative contraindications in unstable patients or in performing intravenous contrast-enhanced CT, such as iodine contrast media allergy or renal failure. Third, interpretation of the results requires significant expertise and the inter-reader agreement is not high.^[32]^ There are many suspicious findings on CT that may complicate the diagnosis. I-FABP is a simple and objective test and might be able to offset weaknesses in CT.

Using the I-FABP test alone or examination alone is insufficient for diagnosing HVI. Surgeons should make a comprehensive assessment that combines physical examination, laboratory, and radiographic findings to diagnose bowel injuries. McNutt et al^[33]^ demonstrated that diagnostic accuracy for bowel injury becomes efficient via scoring using the Bowel Injury Prediction Score (BIPS). The BIPS includes physical examination, presence of leukocytosis, and CT findings. The sensitivity, specificity, negative predictive value, and positive predictive value were 85.7%, 76.2%, 70.6%, and 88.9%, respectively. Similarly, this present study showed that CT findings and physical examinations are useful for diagnosing HVI, but WBC was inconsistent. WBC is known to be unreliable, as previously reported.^[2,6]^ Using I-FABP instead of WBC, the BIPS might be able to diagnose HVI more accurately.

The present study has several limitations. First, even though the study was conducted over 5 years, the number of patients with HVI was small. This could have magnified the effect of selection bias. It is difficult to increase the sample size because blunt HVI is rare.^[19]^ Additionally, we could not analyze a diagnostic scoring system and perform a multivariate analysis because of concerns about the sample size. Multi-institutional studies will be useful in resolving this limitation. Second, physical examinations are more subjective compared to other tests. Many studies reported that physical examination is one of the most important factors for decision-making in emergency abdominal surgery. However, some studies reported that physical examinations have poor interobserver agreement (K value = 0.4–0.7).^[34–36]^ Therefore, several surgeons at our hospital carefully performed a physical examination. Third, this study did not take into account the time and amount of bowel injuries. Biomarker levels may correlate with the amount of bowel injuries and time. Currently, many biomarkers are available at trauma centers, and each biomarker has its distinctive characteristics (e.g., WBC increases when collecting serial samples after admission).^[6]^ However, I-FABP might have a peak at admission.^[13–15]^ Serial I-FABP test is required to diagnose HVI.

## 5 Conclusions

This is the first study to demonstrate the diagnostic value of a biomarker for HVI. I-FABP has a higher negative predictive value compared to traditional diagnostic tests. However, the accuracy of I-FABP alone was insufficient to diagnose HVI in abdominal blunt trauma patients. Surgeons should make a comprehensive assessment. The combination of I-FABP and physical examination may be able to rule out HVI without requiring a CT scan.

## Acknowledgements

The authors thank K. Sekine for invaluable comments and suggestions, H. Funaoka for useful comments and encouragement, and K. Hayashida for providing advice on the analyzed data.
